# A survey of explosive traces in public places

**DOI:** 10.1111/1556-4029.70042

**Published:** 2025-05-07

**Authors:** Richard J. Winder, Samuel A. J. Wilby, Lauren Lessey, Hazel E. Hutson, Sharon M. Broome, Matthew S. Beardah

**Affiliations:** ^1^ Dstl Porton Down Salisbury Wiltshire UK

**Keywords:** background survey, explosive prevalence, forensic explosives, high explosives, inorganic explosives, prevalence, trace explosives

## Abstract

Interpretation and evaluation of trace explosives evidence requires practitioner understanding of factors including transfer, persistence, and environmental prevalence. This study builds on previous work and studies the contemporary prevalence of organic high explosives and inorganic ions of explosives significance in public places. 450 swab and vacuum samples were collected from across Great Britain. Analysis was conducted using liquid chromatography–high resolution mass spectrometry and ion chromatography–mass spectrometry to screen for a wider number of explosives analytes with a higher degree of selectivity and with lower limits of detection than previous studies. Analytes screened for included military high explosives, organic peroxide explosives, and inorganic ions of explosives significance. Only eight low nanogram level traces of organic explosives (HMX, NG, PETN, and RDX) were detected. The results indicate that high explosives traces remain uncommon in the public environment and transport network. Due to the low prevalence, these results strengthen the association between the detection of a trace and explosives activity, and assist the practitioner in assigning significance. Many inorganic ions (ammonium, calcium, chloride, magnesium, nitrate, nitrite, potassium, sodium, and sulfate) were detected at milligram or sub‐milligram quantities. They are common in the environment, naturally occurring, and used commercially. Interpreting the general significance when detecting traces of common inorganic species is challenging. Barium, chlorate, perchlorate, strontium, and thiocyanate were not detected and are therefore more uncommon, strengthening the association between detection and explosives activity.

**Highlights**

- 450 samples collected from public locations are analyzed for organic/inorganic explosives traces.
- Data investigating the prevalence of organic peroxide explosives were obtained for the first time.
- Organic high explosives traces remain uncommon in the public environment.
- Most inorganic ions related to explosives are common, as expected from natural occurrence.
- Chlorate, perchlorate, barium, strontium, and thiocyanate traces are uncommon in public locations.

## 1 INTRODUCTION

Explosives are compounds which are capable of rapid chemical reaction, resulting in deflagration or detonation in response to stimuli such as impact, friction, heat, or a shockwave [1]. By their nature, explosives can cause significant physical damage, injury, and/or death. While they have legitimate uses such as military, demolition, firearms, special effects, and fireworks displays, they are also used by criminals and terrorists [2]. Therefore, manufacture, storage, and use of explosives by members of the public is generally illegal in Great Britain (GB) [3, 4] and many other countries. There are exceptions to this, such as for certain fireworks or licensed gun owners.

To assist police with investigations into the criminal misuse of explosives, operational analysis can either be undertaken on bulk or trace explosives material. Bulk material is visible to the naked eye, typically milligram quantities or higher, although lower quantities could be visible if contained in a small area such as a single crystal. It includes evidence collected pre‐explosion such as from a bomb factory or failed explosive device. Trace explosive residues are invisible to the naked eye, typically nanogram or microgram quantities, although larger quantities may also be invisible if spread over a wide area. These may be recovered post‐explosion where the bulk material has been consumed and, alternatively, pre‐explosion, transferred inadvertently and unknowingly by suspects handling bulk explosive material. Regardless, it is imperative that trace explosives evidence is sufficiently robust to be presented and scrutinized in a court of law.

In addition to forensic scientists having the suitable training and expertise to conduct and interpret physical examinations and chemical analyses, evaluating the significance of explosives trace evidence relies heavily on knowledge of transfer, persistence, and environmental prevalence [5, 6, 7]. Although the recovery of trace explosives residue post‐explosion is unlikely to be contentious, there is a strong requirement to understand explosives significance through comparison of operational results to background levels prevalent in the general environment. Trace results in the pre‐explosion context can be more challenging for the forensic scientist to interpret; knowledge of background levels becomes more important to test hypotheses relating to contamination.

Studies to assess the environmental prevalence of explosives related chemical species in the general environment have previously been published in the UK and abroad [8, 9, 10, 11]; however, these are now decades out of date. A more recent complementary study was completed, covering specifically inorganic ions on hands [12]. Over time, the threat picture has changed. Explosives have continued to be used legitimately for military training, industrial applications, mining, pyrotechnic displays, and illegitimately by criminals and terrorists. Stricter government legislation has limited access to precursor chemicals [13], and there has been continued use of improvised or novel explosives. These bring new challenges to trace analysis, requiring processes capturing a wider range of analytes with differing chemistries.

Limits of detection for the organic explosives in previous studies are given in the range 2–9 ng for “dirty” samples, such as those recovered from typical public surfaces. This estimate does not factor in losses resulting from sample extraction and clean‐up, which were not investigated. With improved instrumentation and sample preparation, sub‐nanogram detection limits are now achievable for some analytes even after losses from extraction and clean‐up are taken into consideration.

The aim of this study was to provide a present‐day survey of environmental levels of explosives in public places across Great Britain for the purpose of assigning significance to operational data analysis and reporting. Analytical instrumentation advancements now facilitate the screening of a wider range of chemical species at lower, in some cases sub‐nanogram, detectable levels. This supports the explosives expert in providing reliable, evidence‐based assessments to assist the court in understanding the significance of their findings.

## 2 MATERIALS AND METHODS

### 2.1 Anti‐contamination

The potential for contamination must be considered when collecting, analyzing, and evaluating the results of trace explosive evidence. Contamination has the potential to occur at all stages of the process: pre‐collection at the scene, during transport, and during analysis. Robust sampling kits and anti‐contamination procedures are required to control this potential, as was applied in this study. At the Forensic Explosives Laboratory (FEL, Wiltshire, United Kingdom), sampling kits are produced in‐house from consumables sourced in a controlled manner to minimize their likelihood of contamination. The kits undergo quality assurance testing once assembled to prove they are free from contamination [9]. Assembly of kits and analysis of trace evidence are performed in a suite of dedicated laboratories with robust anti‐contamination and cleaning procedures. The laboratories are routinely monitored for explosive traces to demonstrate the effectiveness of these procedures [14, 15, 16].

### 2.2 Sample collection

Samples were collected between May 2021 and October 2021.

Sample locations were spread across several towns/cities within Great Britain. The types of locations sampled in each of these towns/cities were public areas/transport with high footfall; a list is provided in Table 1. Surfaces at these location types come into contact with a large number of people and therefore have many opportunities for analyte transfer.

**TABLE 1:** Types of locations, number of sites/samples collected and number of samples containing organic high explosives.

Location type	Number of sites sampled	Location	Total number of samples	No. positive samples, organic high explosives (explosive, mass)
Airport	3	1× Bristol, 2× London	30	1 (PETN 16.3 ng)
Aeroplane	9	3× Bristol, 6× London	54	2 (HMX 1.2 ng, PETN 3.8 ng)
Bus	10	3× Oxford, 4× Swindon, 3× London	70	‐
Hotel room	11	2× Glasgow, 3× Bristol, 2× Newcastle, 2× Nottingham, 2× London	58	1 (RDX 20.1 ng)
Stadium	5	Bristol, Glasgow, Manchester, Newcastle, London	31	–
Taxi	10	3× Cardiff, 3× London, 4× Swindon	58	–
Town/Shopping centre	9	Manchester, Cardiff, Bristol, Exeter, Glasgow, Neilston, Newcastle, Sheffield, London	60	1 (NG 5.3 ng)
Train/Underground train	9	9× London	54	3 (NG, 0.8 ng, 1.8 ng, 0.6 ng)
Train/Underground station	5	1× Exeter, 3× London, 1× Sheffield	35	–
TOTAL	71	–	450	8 (1.8%)

The specific objects and surfaces sampled were dependent on the location. Swab samples were collected from hard, non‐porous surfaces with a particular focus on common touch points, such as handrails, keypads, plastic/metal seats, street furniture, and push‐buttons. Vacuum samples were collected from porous surfaces such as carpets, upholstered seats, and beds. Where the same location type was sampled repeatedly, samples were collected from the same types of surface. For example, highly comparable surfaces were sampled across all 11 hotel rooms because very similar objects are present in all locations, but there was greater variation across town centers where different types of street furniture are used.

The sampling area was not specified because of the variation in size/shape of surfaces. The swab samples with the smallest area were collected from 3 to 4 light switches as a composite (hotel room) and the largest area from rows of metal seating (airport). The vacuum samples with the smallest area were collected from a chair and the largest area from carpet (≈8 m^2^).

Permission was obtained from the relevant parties prior to the collection of samples. All locations were sampled during or after a period of normal usage, before any routine cleaning, to ensure a representative sample. An approximate time since the location was last cleaned was requested and recorded where known; this was variable from 1 day to greater than 1 year. However, even where a location had been more recently cleaned, it was not possible to determine exactly which surfaces or types of cleaning products were used. Due to the COVID‐19 lockdown, enhanced cleaning measures were in place in many locations compared with pre‐COVID‐19. Alongside sample details, approximate temperature, weather, and whet indoors or outdoors, if not obvious from location, were recorded.

Sampling was carried out by two operators working together; one of these operators remained the same across all locations to ensure consistency.

### 2.3 Sampling equipment

All equipment used for sampling was assembled in a dedicated trace explosives laboratory, which was subject to regular environmental monitoring to confirm it was free from explosives traces.

Prior to removal from the laboratory, 1 in 40 sampling kits underwent quality assurance testing to ensure they were free from detectable organic explosives contamination prior to use, as previously detailed [9]. Limits of detection for quality assurance were equivalent to those for sampling, typically sub‐nanogram. The equipment was sealed in multiple layers of packaging, which were subsequently removed when loading the equipment into and out of the vehicle to reduce the chance of contaminating the sampling location prior to sampling.

Before entering a sampling location, the samplers' hands were cleaned with isopropyl alcohol (IPA) wipes. Gloves, a Tyvek suit, and a surgical mask were donned to reduce the chance of the sampler contaminating the sample location.

#### 2.3.1 Swab sampling

**FIGURE 1:**
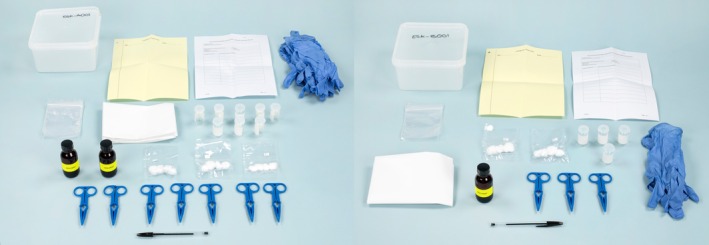
Contents of the swab sampling kit ESK‐A (left) and ESK‐B (right).

In‐house swab sampling kits (environmental sampling kit, ESK) contained glazed paper, nitrile gloves, 50:50 Ethanol (AnalaR, Fisher):Water (AnalaR, Fisher), disposable forceps, cotton swabs, 10 mL soda glass vials, grip seal bags, pen, and paper in a plastic container sealed in nylon packaging, as shown in Figure 1. Two types of kits were prepared, ESK‐A and ESK‐B. Each ESK‐A kit could sample up to 6 surfaces (+ 2 control samples). Each ESK‐B kit could only sample up to 2 surfaces (+ 2 control samples) as it was intended to be used alongside a vacuum sampling kit.

To swab a surface, a quantity of 50:50 ethanol:water was dispensed into a soda glass vial. Using the disposable forceps, the swab was wetted from the soda glass vial and moved backward and forward over the surface. Once collected, the swab was sealed in the soda glass vial with the remaining solvent, and the forceps were disposed of. Before collecting samples, a swab/solvent control was obtained by placing a swab directly into a soda glass vial containing the 50:50 ethanol:water; this was to demonstrate the kit had not become contaminated during storage/transport. An operator control was also collected in the same manner as a sample, by swabbing the gloves and Tyvek suit of the operator after donning; this was done to demonstrate the operator had not introduced contamination to the sampling location.

#### 2.3.2 Vacuum sampling

**FIGURE 2:**
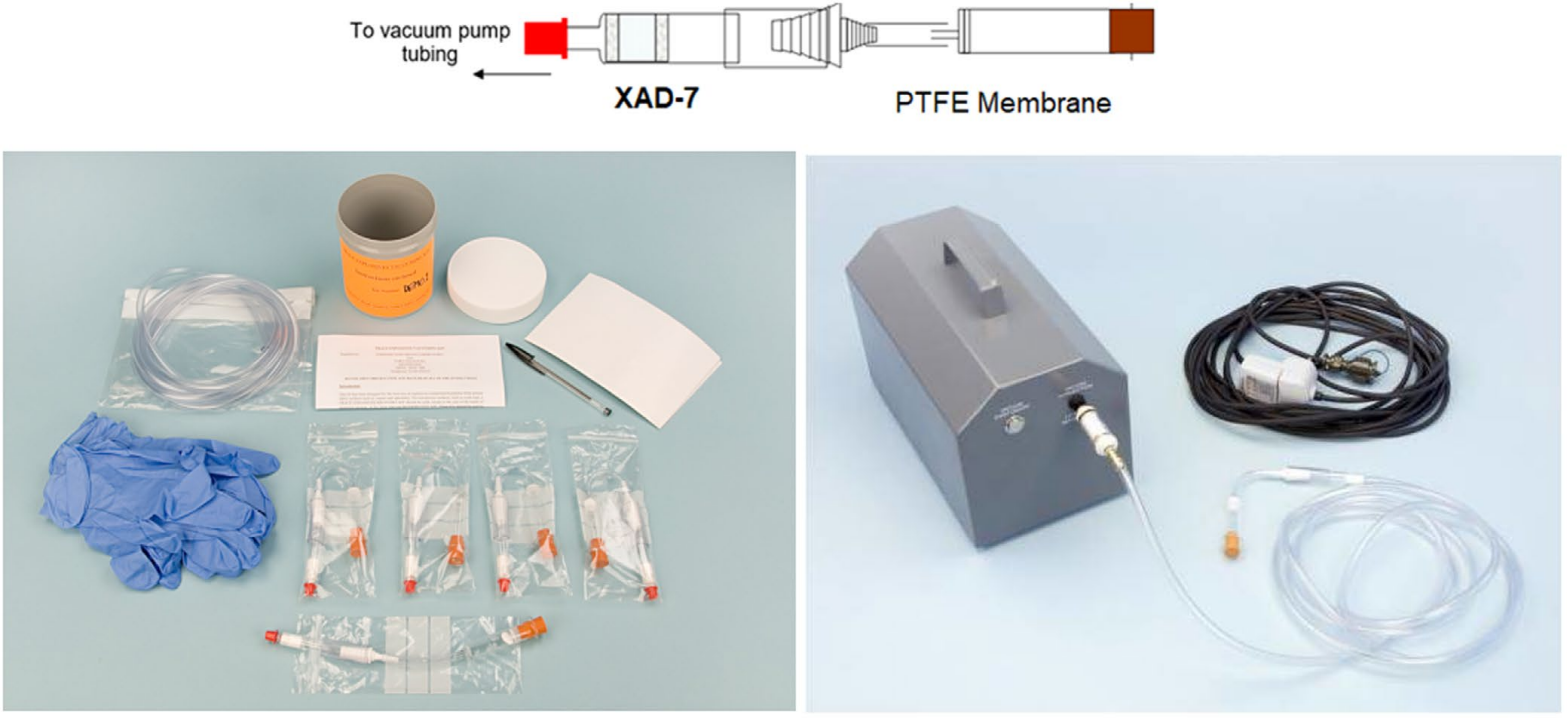
(Top) Diagram of the vacuum sampling assembly, (Bottom Left) contents of the vacuum sampling kit, and (Bottom Right) vacuum pump.

In‐house vacuum sampling kits (trace explosives vacuum kit, TEVK) contained glazed paper, nitrile gloves, pen, paper, vacuum sampling assembly (glass syringe barrel with polytetrafluoroethylene (PTFE) filter membrane in line with XAD‐7 sorbent tube) and PTFE tubing to connect to the vacuum pump, as shown in Figure 2. XAD‐7 is a commercial hydrophobic organic porous polymer that can be purchased in glass sorbent tubes for headspace analysis. Each TEVK could sample up to 5 surfaces (+ 1 control sample). The vacuum pump used in this work was designed in‐house with cleanable surfaces and minimized crevices to ensure it trapped minimal contamination and could undergo suitable decontamination between locations. It has a nominal flowrate of 12.7 lpm @ 600 mbar.

To vacuum a surface, the operator connected the vacuum sampling assembly to the vacuum pump, removed the rubber bung, turned on the pump, and moved the inverted syringe barrel assembly over the surface at an angle to agitate any debris for a minimum of 2 min, or until the surface area was fully sampled. The rubber bung was replaced in the end of the syringe barrel after sample collection to retain the collected material. Prior to sampling, a vacuum control sample was taken to demonstrate the kit had not become contaminated during storage/transport. Vacuum sampling was always combined with swab sampling, so no additional vacuum sampling operator control was required.

#### 2.3.3 Post‐sampling

Once collected, samples were re‐sealed in their respective kit box, which was further sealed in nylon packaging to prevent contamination.

If more than one location was sampled in the same day, the whole process was repeated, including the sampler wiping hands and donning new gloves, Tyvek suit, and surgical mask, to prevent cross‐contamination; new control samples were also taken.

### 2.4 Sample processing and analysis

Samples were processed in a dedicated trace explosives laboratory according to standard laboratory procedures. This was achieved by manually pounding the swab with a clean glass pipette to extract the analytes into the ≈5 mL residual solvent. Solvent was removed to a separate soda vial; a further 5 mL 50:50 ethanol:water was added, and the swab was pounded again. The second aliquot was combined with the first to ensure maximum recovery.

A 0.5 mL aliquot of the combined extract was removed for inorganic analysis; this was diluted with 0.5 mL of 18.2 MΩ ultrapure water to dilute the ethanol and matrix prior to analysis. The remainder of the combined extract was subjected to a clean‐up procedure to remove the sample matrix and improve the limit of detection through sample concentration. Solid phase extraction (SPE) sorbent (Biotage ENV+ 1000 mg) was used, and the target analytes were eluted into 1 mL Acetonitrile (Fisher) for organic high explosive analysis. Internal validation studies have shown an SPE recovery efficiency of between 10% (Nitrotoluene) and 58% (Trinitrotoluene, TNT) with an average across all analytes of 32% (σ = 15%). The reduction from 10 mL sample extract to 1 mL SPE eluent concentrated the sample to improve the limit of detection. For all analytes except triacetone triperoxide (TATP), hexamethylene triperoxide diamine (HMTD), and 2,3‐dimethyl‐2,3‐dinitrobutane (DMDNB), an additional 10x concentration was achieved by evaporating 500 μL of solution to 50 μL under a stream of nitrogen gas.

A multi‐component positive control was cleaned up alongside every batch of samples to demonstrate the procedure had been carried out correctly. The positive control multi‐component standard solution was spiked onto a swab or vacuum tube assembly, extracted, and processed identically to the samples.

Analysis for organic high explosives was carried out using liquid chromatography high resolution mass spectrometry (LC‐HRMS), according to ISO17025 accredited in‐house laboratory methods, using a Thermo‐Scientific Dionex Ultimate 3000/Q‐Exactive+. Analysis for cyclotetramethylene tetranitramine (HMX), ethylene glycol dinitrate (EGDN), cyclotrimethylene trinitramine (RDX), diethylene glycol dinitrate (DEGDN), nitroglycerine (NG), dinitrotoluene (DNT), diazodinitrophenol (DDNP), erythritol tetranitrate (ETN), TNT, pentaerythritol tetranitrate (PETN), picrate, nitrobenzene (NB), tetryl, pentitol pentanitrates (such as xylitol pentanitrate (XPN)), and hexitol hexanitrates (such as sorbitol hexanitrate (SHN) and mannitol hexanitrate (MHN)) was carried out using an ACE C18‐Ar (150 × 2.1 mm i.d., 2 μm) with an injection volume of 2 μL and methanol:water gradient elution. The eluent contained 0.5% chloroform and detection used atmospheric pressure chemical ionization (APCI) with positive/negative switching. Instrument run time was 27 min plus 9 min equilibration time. Analysis for HMTD, DMDNB, TATP, hexamethylene diperoxide diamine (HMDD), diacetone diperoxide (DADP), and methyl ethyl ketone peroxide (MEKP) was carried out using a YMC C18 Triart column (50 mm × 2.0 mm id., 1.9 μm) with an injection volume of 1 μL and methanol:water gradient elution. The eluent contained 6 mmol ammonium acetate and detection used APCI in positive mode. Instrument run time was 9.5 min plus 11.5 min equilibration time. For both methods, identification was by comparison of retention time and accurate mass to a multi‐component standard produced from certified reference materials (CRM).

Instrumental limits of detection were typically in the range 0.001 ng/L to 0.005 ng/μL, with exceptions for NB (5 ng/μL), TATP (0.5 ng/μL), DDNP/EGDN (0.25 ng/μL), and DEGDN/DNT/ETN/HMTD (0.05 ng/uL).

Analysis for inorganic ions of explosives significance was carried out using ion chromatography mass spectrometry (IC‐MS), according to an in‐house ISO17025 accredited laboratory method, using a Thermo‐Scientific ICS6000/ISQ‐EC. Analytes screened for included ammonium, barium, calcium, chlorate, chloride, magnesium, nitrate, nitrite, perchlorate, potassium, sodium, strontium, sulfate, and thiocyanate. Anions analysis was carried out using an IonPac AS20 (2 × 250 mm) column with an injection volume of 20 μL and potassium hydroxide gradient elution. Cations analysis was carried out using an IonPac CG12A (2 × 250 mm) column with an injection volume of 20 μL and methanesulphonic acid gradient. Detection used a conductivity detector followed by electrospray ionization (ESI) mass spectrometry. IPA was infused before the mass spectrometer at a flow rate between 0.01 and 0.14 mL/min to enable the formation of cation adducts. Identification was by comparison of retention time to a multi‐component standard produced from certified reference materials (CRM) and the presence of two ions with characteristic mass‐to‐charge ratio per analyte. Limits of detection were 0.1 ng/μL except for magnesium (0.5 ng/μL), sulfate/ammonium (1 ng/μL), phosphate (2.5 ng/μL), and calcium (10 ng/μL).

### 2.5 Limitations

It is accepted that the number of samples collected is low relative to the size of Great Britain. The discussion and conclusions reflect this limitation. It is anticipated that more samples will be added to the data over time to increase the size of the data set.

For inorganic ions, anions/cations cannot be associated when analyzed by IC‐MS, for example, if ammonium and nitrate ions are detected in the sample, it does not mean there is ammonium nitrate present.

The detection limits for this study, factoring in sample processing losses and instrumental limits of detection, are shown in Table 2. These provide an estimate of how much material would need to be present within the collected sample in order to produce a positive result. Due to variation in recovery, which is dependent on surface type [17], these values do not account for surface recovery and therefore do not provide an estimate of the quantity of material required to be on the surface prior to sampling.

**TABLE 2:** Approximate limits of detection derived from instrumental limits of detection coupled with sample processing and estimated SPE recovery.

Analyte	Approximate detection limit with clean‐up recovery (ng)
DDNP	278
DEGDN	68
DNT	7
EGDN	263
ETN	93
HMX	0.4
NB	1389
NG	3
NT	500
PETN	0.6
Picric acid	0.5
RDX	0.9
TNT	0.9
DADP	9300
DMDNB	980
HMTD	417
MEKP	4300
TATP	1000
Ammonium	20,000
Barium	50,000
Calcium	200,000
Chlorate	2000
Chloride	2000
Magnesium	10,000
Nitrate	2000
Nitrite	2000
Perchlorate	2000
Potassium	2000
Sodium	2000
Strontium	20,000
Sulfate	20,000
Thiocyanate	2000

## 3 RESULTS AND DISCUSSION

### 3.1 Organic explosives

A total of 450 samples were collected from across Great Britain; results are shown in Table 1. Organic high explosives were detected in a total of 8 samples, including HMX, NG, PETN, and RDX. For each of these detections, the retention time was within ±2% and accurate mass within ±2 ppm of the CRM standard. This equates to a total of 1.8% positive detection rate, suggesting organic high explosives traces are uncommon in the public environment. No explosives were detected in the corresponding blank or negative control samples, suggesting it is highly unlikely that these are false positives as a result of contamination.

There were no instances of detection of improvised explosives that were not included in previous screens, such as organic peroxides (e.g., TATP, HMTD) or sugar nitrates (pentitol pentanitrates, hexitol hexanitrates). These have no legitimate commercial uses and therefore fewer opportunities to enter the public environment. Typically, they are only expected after significant explosive events such as the Manchester Arena bombing. The explosives detected were limited to military/commercial high explosives [18], which may be handled regularly by military personnel, for example, during training. PETN is contained within detonating cord, which has industrial applications such as demolition, mining, and civil works. NG is also used in smokeless powder (firearms propellant) [19] and some heart medication [20] so may also be handled regularly by armed police and select members of the public. Heart medication contains milligram quantities of NG [21]; this does not pose an explosive risk but is well within the limits of detection of this study. As suggested in previous literature [9, 10], these could act as legitimate sources of environmental contamination.

Approximate quantities (in sample) were obtained through single point calibration and ranged from 0.8 to 20.1 ng. These are considered low‐level traces. Instrumental error using single point calibration, determined during method validation for ISO17025 accreditation, is up to ±90% (expanded uncertainty coverage factor *k* = 2). Single point calibration is appropriate for this analysis because its purpose is to understand the frequency of detection with an approximate quantity (e.g., order of magnitude). However, the reported quantities do not include back calculation for sampling or extraction/SPE recovery. The positive control was used to confirm sample extraction/SPE recovery. Analyte recovery was between 34% (HMTD) and 78% (PETN) with an average across all analytes of 56% (σ = 16%); this is marginally higher than the figure obtained during the internal validation study (32%, σ = 15%). Different operators were involved, and operator to operator variation is expected. If extraction/SPE efficiency was factored onto the reported quantities, they would approximately double. Sampling uncertainty is unable to be accurately estimated due to the wide variety of surface types encountered. A study has shown that, for ethanol:water wetted cotton swabs recovery, sampling recovery can be ≈20% to ≈95% [17]. If this was factored into the reported quantities, they would be from 1.05 to 5 times higher. Operationally, variation in sampling recovery is likely to be higher due to additional factors such as surface roughness, surface area, and the presence of dirt.

Organic explosives, such as HMX or TNT, are not naturally occurring and have only limited legitimate use, so they were not expected to be prevalent in the public environment. The percentage of positive samples (1.8%) and approximate quantity (0.8 ng – 20.1 ng) support this assumption. The results remain consistent with previous studies in terms of type of explosive (military/commercial), frequency of detection, and quantity. The first study yielded a positive detection rate of 1.2% with quantities of NG, TNT, PETN, and RDX at quantities from 5 to 19 ng [9], and the second study a positive detection rate of 0.8% with quantities of NG, RDX, and DNT at quantities from 4 to 15 ng [10]. This similarity is observed, despite a wider range of analytes now being screened.

The results imply that organic high explosives traces detected on individuals or their clothing/possessions are unlikely to have originated from contamination in the public environment and therefore imply an association with explosives. Exceptions to this must be considered for NG and PETN. Both are vasodilators and are contained in prescription medication, although PETN‐containing medication is no longer prescribed in the UK [22]. Where an individual is taking such medication, traces may be detected without explosives association.

### 3.2 Inorganic ions of explosives significance

The results for individual inorganic ions are summarized in Table 3; the results were more variable than for organic explosives, which was expected. Unlike organic explosives, many of the ions found in inorganic explosives are naturally occurring and have widespread legitimate use. A small number of the samples that were collected (19/450) were not able to be analyzed for inorganic ions either because the aliquot was not separated before SPE clean‐up, breakage, or leakage of the solution. Therefore, the total number of inorganic samples analyzed was 431.

**TABLE 3:** Results for samples containing inorganic ions of explosive significance.

Ion	Control sample detection rate	Control sample quantity (mg)	Sample detection rate	Sample quantity (mg)
Ammonium	2/321 (<1%)	<0.5 (<LoQ)	41/431 (10%)	<0.5–3
Barium	0/321 (0%)	–	0/431 (0%)	–
Calcium	0/321 (0%)	–	8/431 (2%)	0.1–0.9
Chlorate	0/321 (0%)	–	0/431 (0%)	–
Chloride	23/321 (7%)	<0.01–0.6	409/431 (95%)	<0.01–5
Magnesium	0/321 (0%)	–	60/431 (14%)	<0.01–0.5
Nitrate	29/321 (9%)	0.02–0.16	207/431 (48%)	<0.01–2
Nitrite	0/321 (0%)	–	8/431 (2%)	<0.05 (<LoQ)
Perchlorate	0/321 (0%)	–	0/431 (0%)	–
Potassium	144/321 (45%)	0.02–0.2	419/431 (97%)	<0.01–1
Sodium	302/321 (94%)	0.04–0.5	429/431 (99%)	0.05–2
Strontium	0/321 (0%)	–	0/431 (0%)	–
Sulfate	4/321 (1%)	0.06–0.2	283/431 (65%)	<0.02–10
Thiocyanate	0/321 (0%)	–	0/431 (0%)	–

*Note*: The lower limit of the quantity range is reported as < where at least one sample contained the ion, but below the limit of quantification.

Many ions were detected, some in a very high percentage of samples; these include sodium (99%), potassium (97%), chloride (95%), nitrate (48%), sulfate (65%), magnesium (14%), ammonium (10%), calcium (2%), and nitrite (2%). Sodium (94%), potassium (45%), nitrate (9%), chloride (7%), sulfate (1%), and ammonium (<1%) were also detected in control samples, suggesting their abundance is such that it is difficult to avoid introducing them into the sample from the equipment or sampler. For example, these ions were also found in a high percentage of samples collected from human hands [12].

**FIGURE 3:**
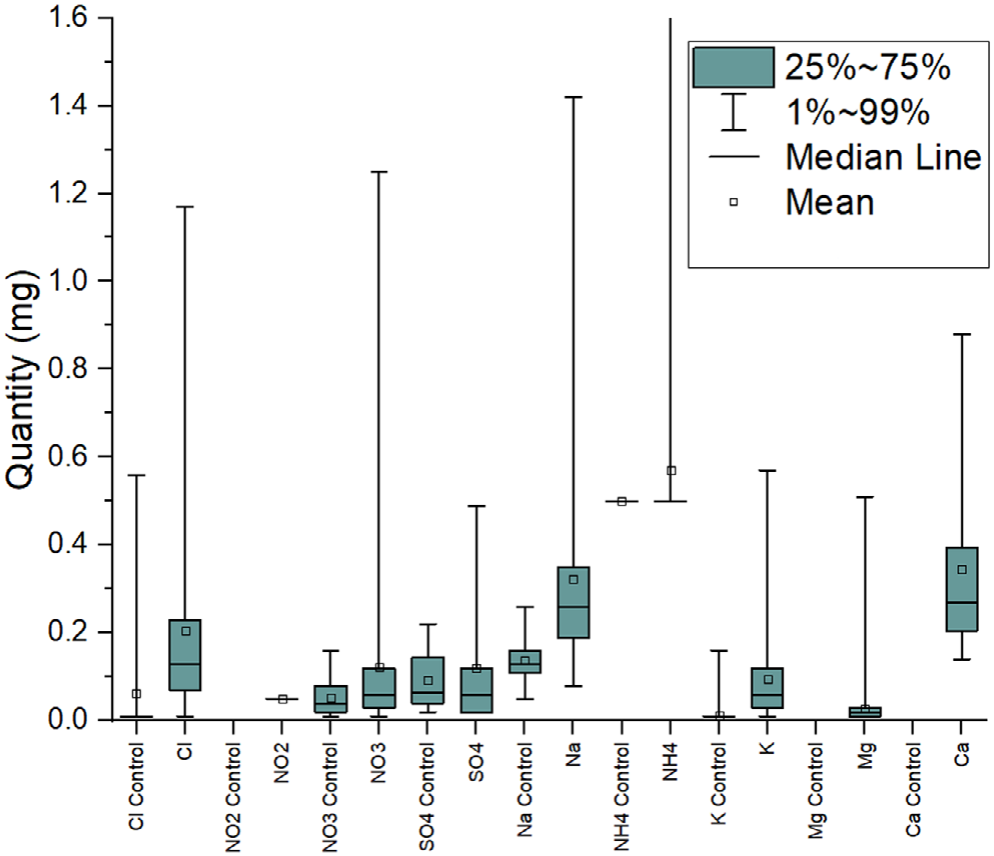
Box plot showing the distribution of quantities of detected inorganic ions. Where the quantity detected was less than the LoQ, the LoQ value was substituted to represent the maximum quantity that could have been present.

The range and distributions of recovered quantities (mg) are shown in Table 3 and Figure 3 alongside the equivalent control ranges/distributions. For all but one of the detected analytes, the 99th percentile is below 1.5 mg; for ammonium, it is 3.4 mg. These quantities are approximately 6 orders of magnitude greater than the recovered quantities of organic explosives. The reason for their greater prevalence and quantity is natural occurrence. Sodium, potassium, and magnesium are some of the most naturally abundant elements in the earth's crust/sea, are present in many rock/mineral formations, and play an important part in biological processes [23]. Sulfate is also naturally occurring in rocks/minerals, and nitrate is widely used as a fertilizer [24, 25]. These ions also have explosive significance; a number of common pyrotechnic compositions include potassium‐ and/or nitrate‐based chemicals, for example, potassium nitrate is a component of black powder [1, 7]. Magnesium may be used as a fuel in pyrotechnic formulations or used to ignite thermite [26]. Sulfate and chloride have limited explosive significance but may be present in post‐explosion residue from sulfur or chlorate containing explosives.

These results reduce the significance of low milligram level trace detection of the 9 common ions recovered, suggesting limited correlation between the presence of these ions and association with explosives at the quantities detected in these environmental samples. However, elevated levels (e.g., orders of magnitude above those detected here) could still be considered significant post‐explosion, given their association with the physical evidence of an explosive event.

Barium, chlorate, perchlorate, strontium, and thiocyanate were not detected in any samples, so they are suggested as uncommon in the public environment. Barium and strontium salts are used to give color to pyrotechnic formulations [27]; barium is used in primer compositions [28]; chlorate and perchlorate are used as oxidizers in explosives and pyrotechnic formulations (e.g., chlorate:sugar, flash powder) [29]; and thiocyanate is used in some primer compositions and electric matches [27, 30, 31]. These results increase the significance of trace detection of these 5 uncommon ions, suggesting a correlation between the presence of these ions and an association with explosives. However, natural occurrence and legitimate non‐explosive uses must still be considered within the context of a particular case. For example, barium sulfate is used as a filler within paints [32], so it may be more likely to be detected when swabbing certain types of painted surfaces.

While some generalized inferences can be made relating to both organic and inorganic ions, forensic trace interpretation must be carried out on a case‐by‐case basis, using not only the fundamental data obtained as part of this study to understand the prevalence within public locations, but also the specific circumstances of the case. This includes knowledge of the activities at the scene, the sample collection process, and any other relevant information.

## 4 CONCLUSIONS

As part of a criminal investigation relating to the misuse of explosives, swabs and/or vacuum samples may be collected and analyzed for explosives traces, for example, for the purpose of linking suspects, places, and objects to an explosion or bomb factory. Interpretation of trace explosives evidence requires, amongst other things, knowledge of the prevalence of the analytes in question. The lower the prevalence, the greater the significance of a positive finding in the context of a case. It is possible that certain activities, for example, commercial use, military training, legitimate firearms use, and terrorist attacks, may lead to a greater prevalence of explosives analytes in the public environment. The prevalence of analytes should be monitored periodically to provide practitioners with data to undertake interpretation of results.

While environmental surveys for explosives traces have previously been undertaken, 20 years have elapsed, so the data are no longer contemporary and new data were required. More recent complementary studies have focused on hands, but only covered inorganic ions [12]. Furthermore, a larger number of organic explosives analytes are now detectable at trace levels, for which no data were available. In this study, 450 swab/vacuum samples were collected from public places and across the transport network for the purpose of providing contemporary data covering many organic and inorganic analytes. The results from vacuum samples were not directly compared with swab samples due to collection from different surface types, for example, hard surfaces vs. soft surfaces. However, positive results were obtained from both sampling methods, demonstrating their suitability for explosive recovery.

From the 450 samples, traces of HMX, NG, PETN, and/or RDX were detected in 8 samples (1.8% of total samples); individual samples contained approximately 0.8–20 ng of explosive. This suggests that organic high explosives are uncommon in the public environment, and that where present these are at very low levels. Due to the low prevalence, these results suggest that contamination of an individual or their clothing/possessions from the public environment is unlikely, this strengthens their association with explosives where such traces are detected. Although this study incorporates a wider number of organic high explosive analytes than previous work [9, 10], the percentage of positive samples, 1.8%, is similar and the types of explosives detected were also military/commercial high explosives. General consistency with previous studies despite a wider number of target analytes, improved selectivity, and sub‐nanogram limits of detection for some analytes, is encouraging and suggests stability of background levels over time.

The results for inorganic ions related to explosives are more variable than organic high explosives; this was expected and is a result of their natural occurrence and widespread legitimate use. Many ions were detected, some in a very high percentage of samples; these include sodium (99%), potassium (97%), chloride (95%), nitrate (48%), sulfate (65%), magnesium (14%), ammonium (10%), calcium (2%), and nitrite (2%). Sodium, chloride, nitrate, sulfate, and potassium were also detected in some control samples. Conversely, chlorate, perchlorate, thiocyanate, barium, and strontium were not detected in any samples or controls. These results indicate that common inorganic ions are ubiquitous in the public environment, and detection of low trace quantities is of limited explosives significance when interpreted generally, in the absence of case‐specific information. Detection of chlorate, perchlorate, barium, strontium, and thiocyanate, which are uncommon, may be of greater significance because of their low prevalence. The results of this study provide a greater understanding of their detection rates. Approximate levels provide useful information to aid interpretation in more specific scenarios, for example, when detected post‐explosion at elevated levels compared to those reported here, for example, hundreds of milligrams, and with corresponding physical evidence.

## CONFLICT OF INTEREST STATEMENT

The authors have no conflicts of interest to report.

## References

[1] Meyer R , Kohler J , Homburg A . Explosives. 7th ed. Weinheim, Germany: Wiley‐VCH Verlag GmbH & Co. KGaA; 2016. p. 117–140.

[2] Klapec DJ , Czarnopys G , Pannuto J . Interpol review of the analysis and detection of explosives and explosives residues. Forensic Sci Int Synerg. 2023;6:100298. 10.1016/j.fsisyn.2022.100298 36685733 PMC9845958

[3] HMSO . Explosives Act 1875, (38 and 39 Vict c.17). London, UK: HMSO; 1875.

[4] The Stationery Office . Explosives Regulations 2014, SI 2014/1638. London, UK: The Stationery Office; 2014.

[5] European Network of Forensic Science Institutes . Best practice manual for the forensic recovery, identification and analysis of explosives traces. Report No.: ENFSI‐BPM‐EXP‐01. Wiesbaden, Germany: European Network of Forensic Science Institutes; 2015.

[6] European Network of Forensic Science Institutes . ENFSI guideline for evaluative reporting in forensic science. Wiesbaden, Germany: European Network of Forensic Science Institutes; 2016.

[7] Murray GT . The signficance of analytical results in explosives investigation. In: Beveridge A , editor. Forensic investigation of explosions. 2nd ed. Boca Raton, FL: CRC Press; 2012.

[8] Walker C , Cullum H , Hiley R . An environmental survey relating to improvised and emulsion/gel explosives. J Forensic Sci. 2000;46(2):254–267. 10.1520/JFS14958J 11305427

[9] Crowson A , Cullum H , Hiley R , Lowe A . A survey of high explosives traces in public places. J Forensic Sci. 1995;41(6):980–989. 10.1520/JFS14035J 15317181

[10] Cullum H , McGavigan C , Uttley C , Stroud M , Warren D . A second survey of high explosives traces in public places. J Forensic Sci. 2004;49(4):684–690. 10.1520/JFS2003237 15317181

[11] Lahoda KG , Collin OL , Mathis JA , LeClair HE , Wise SH , McCord BR . A survey of background levels of explosives and related compounds in the environment. J Forensic Sci. 2008;53(4):802–806. 10.1111/j.1556-4029.2008.00743.x 18537869

[12] van Damme IM , Hulsbergen AWC , Allers S , Bezemer KDB , Miller JV , van Asten AC . A study into the natural occurrence of inorganic ions relevant to forensic explosives investigations on human hands. Forensic Sci Int. 2024;361:112119. 10.1016/j.forsciint.2024.112119 38917507

[13] The Stationery Office . Poisons Act 1972, c.66, Sch 1A, Pt. 1. London, UK: The Stationery Office; 1972.

[14] Crowson A , Hiley RW , Todd CC . Quality assurance testing of an explosive trace analysis laboratory. J Forensic Sci. 2001;46(1):53–56. 10.1520/JFS14910J 11210923

[15] Crowson A , Cawthorne R . Quality assurance testing of an explosives trace analysis laboratory – further improvements to include peroxide explosives. Sci Justice. 2012;52(4):217–225. 10.1016/j.scijus.2012.07.001 23068772

[16] Crowson A , Doyle SP , Todd CC , Watson S , Zolnhofer N . Quality assurance testing of an explosives trace analysis laboratory‐further improvements. J Forensic Sci. 2007;52(4):830–837. 10.1111/j.1556-4029.2007.00464.x 17524053

[17] Song‐im N , Benson S , Lennard C . Evaluation of different sampling media for their potential use as a combined swab for the collection of both organic and inorganic explosive residues. Forensic Sci Int. 2012;222(1):102–110. 10.1016/j.forsciint.2012.05.006 22658743

[18] Mathieu J , Stucki H . Military high explosives. Chimia (Aarau). 2004;58(6):383. 10.2533/000942904777677669

[19] Roberts M , Petraco N , Gittings M . Novel method for the detection of nitroglycerin in smokeless powders. Sci Justice. 2015;55(6):467–471. 10.1016/j.scijus.2015.08.001 26654082

[20] Marsh N , Marsh A . A short history of nitroglycerine and nitric oxide in pharmacology and physiology. Clin Exp Pharmacol Physiol. 2000;27(4):313–319. 10.1046/j.1440-1681.2000.03240.x 10779131

[21] Mylan Products Ltd . Package leaflet: Minitran 5 mg or 10 mg transdermal patches. 2024 [accessed 2025 Jan]. Available from: https://www.medicines.org.uk/emc/files/pil.9515.pdf

[22] Pentaerythritol tetranitrate long term trends [database on the Internet]. Bennett Institute for Applied Data Science, University of Oxford. [accessed 2025 Jan]. Available from: https://openprescribing.net/long_term_trends/

[23] Haynes WM . CRC handbook of chemistry and physics. 97th ed. Boca Raton, FL: CRC Press; 2016.

[24] Dawson CJ , Hilton J . Fertiliser availability in a resource‐limited world: production and recycling of nitrogen and phosphorus. Food Policy. 2011;36:S14–S22. 10.1016/j.foodpol.2010.11.012

[25] Anthony JW , Bideaux RA , Bladh KW , Nichols MC . Handbook of minealogy. Chantilly VA: Mineralogical Society of America; 2003.

[26] Klein O . An improved method for igniting thermite reactions. J Chem Educ. 1937;14(7):320. 10.1021/ed014p320

[27] Conkling JA , Mocella C . Chemistry of pyrotechnics: basic principles and theory. 3rd ed. Boca Raton, FL: CRC Press; 2018.

[28] Charles S , Nys B , Geusens N . Primer composition and memory effect of weapons – some trends from a systematic approach in casework. Forensic Sci Int. 2011;212(1–3):22–26. 10.1016/j.forsciint.2011.05.001 21620596

[29] Horrocks AJ , Detata D , Pitts K , Lewis SW . Chlorate‐based homemade explosives: a review. WIREs Forensic Sci. 2024;6(2):1506. 10.1002/wfs2.1506

[30] Lake E . Percussion primers, design requirements. Revision A. St. Louis, MO: McDonnel Aircraft Company; 1976; Report No.: MDC A0514.

[31] Son S , Hiskey M , Naud D , Busse J , Asay B . Lead‐free electric matches. Proceedings of the 29th International Pyrotechnics Seminar; 2002 July 14–19; Westminster, CO. Gilbert, AZ: International Pyrotechnics Society; 2002.

[32] Charles S , Bui DW , Canler T , Carnevali A . Strontium in barium sulfate as a discriminating factor in the forensic analysis of tool paint by SEM/EDS. Forensic Sci Int. 2022;331:111127. 10.1016/j.forsciint.2021.111127 34894612

